# Prevalence and Correlates of Substance Abuse Among Healthcare Students

**DOI:** 10.1155/tswj/5597067

**Published:** 2025-06-02

**Authors:** Rania Mansour Magableh, Amjad Hasan Bazzari, Firas Hasan Bazzari, Ferial Ahmad Hayajneh

**Affiliations:** ^1^Department of Pharmaceutical Sciences, School of Pharmacy, The University of Jordan, Amman, Jordan; ^2^Department of Basic Scientific Sciences, Faculty of Science, Applied Science Private University, Amman, Jordan; ^3^Department of Pharmacy, Faculty of Pharmacy, Jerash University, Jerash, Jordan; ^4^Department of Clinical Nursing, School of Nursing, The University of Jordan, Amman, Jordan

**Keywords:** addiction, drug abuse, drug misuse, healthcare students, undergraduate

## Abstract

Substance abuse among healthcare students is a growing issue across various regions, including the Middle East. Here, we investigate its prevalence and correlates in Jordan. The study utilized an online questionnaire consisting of demographics, attitudes toward substance abuse, the Drug Abuse Screening Test (DAST-10), and the prevalence and types of substances of abuse reported by the participants. A total of 465 students participated, including males (41.3%) and females (58.7%). The results revealed a high prevalence of substance abuse (13.76%), which correlated with DAST-10 scores (*ρ* = 0.442, *p* < 0.01) and was associated with experiencing suicidal thoughts (*p* < 0.01). Higher odds (*p* < 0.05) for substance abuse were observed with smoking (OR = 1.81), working (OR = 2.02), attending private universities (OR = 2.03), studying pharmacy compared to medicine, dentistry, and nursing (OR = 4.85) and being a second year student (OR = 3.23). However, it was not associated with gender, age, marital status, living arrangement, attended high school, GPA, and attending a course covering CNS drugs. In terms of attitudes, substance abuse was associated (*p* < 0.05) with the following: not believing that substance abusers should be punished or that childhood or friends contribute to substance abuse, believing that abusers cannot fully recover, and still wanting to interact with a healthcare provider who has a history of substance abuse. Lastly, seven drugs of abuse classes were identified, and the main reported class was benzodiazepines (22.6%). In conclusion, substance abuse is prevalent among healthcare students in Jordan and is associated with various demographic and attitude factors, which should be taken into consideration for developing interventional and preventative strategies to mitigate this issue.

## 1. Introduction

Substance abuse is an emerging issue among healthcare students, which requires careful examination to reveal its determinants and assess its implications [1–3]. Generally, drug abuse is defined as “the intentional, nontherapeutic use of a drug product or substance, even once, to achieve a desired psychological or physiological effect” [4]. Achieving euphoria, induction of hallucinations, and altering cognition and mood, among others, are all examples of the sought desired psychological effects by substance abusers [5].

Healthcare schools' curricula, from different fields of study, can be described as rigorous and relatively more challenging and demanding than other sectors. This, in turn, may be a significant contributing factor to the increased incidence of anxiety, stress, agitation, sleep disturbances as well as behavioral changes, and the development of depressive symptoms among the students [6]. Such findings were linked to the increased risk of substance abuse as a coping mechanism to ease stress, burnout, and other symptoms [7, 8].

In the Middle East, recent reports revealed concerning patterns of substance abuse among healthcare students. For instance, in a sample of university students in Egypt, medical students were found to have the highest percentage of drug-related problems [3]. Moreover, in Palestine, substance abuse rates were found to be associated with escapism and stress, especially during the exam period [9]. As substance abuse among healthcare students can negatively affect their judgment, educational achievement, and clinical performance [10, 11], addressing the issue of substance abuse is critical, given their future roles as healthcare providers and the potential impact on their well-being and patient safety [12].

In Jordan, commendable efforts were undertaken to mitigate substance abuse; nevertheless, there are gaps in assessing and understanding the issue within the scope of university students and the healthcare system. This was noted in the 2023 report by the Jordanian Anti-Narcotics Department, which highlighted the alarming increase in drug abuse incidents and the need for further cooperation between all relevant ministries and institutions to foster a culture that combats substance abuse and associated crimes [13]. In response, the Jordanian Ministry of Health has put forth a 4-year national action plan to deal with mental health and substance use, which signified the need for comprehensive data on substance abuse among various population groups, including students and healthcare workers [14]. This is in addition to designing and implementing awareness initiatives on substance use targeting healthcare providers and students in healthcare disciplines [14]. From an international perspective, the World Health Organization (WHO) has focused in its 2024 regional assessment on the necessity of country-specific studies investigating substance abuse-related issues and enhancing effective prevention strategies [15]. Therefore, identifying the factors that may influence substance abuse among various population groups, including university students, and planning interventional strategies for high-risk groups can all significantly contribute to the protection and improvement of the population's well-being [16].

From the aforementioned, this study is aimed at investigating the prevalence and associated factors related to substance use among healthcare students in Jordan, which addresses the gap by providing insights into the matter and aiding in developing future targeted interventions to mitigate the risks and promote better practices within this critical group.

## 2. Methods

### 2.1. Sampling and Ethical Considerations

The study was conducted using an online questionnaire in English, developed via Google Forms, that was distributed among Jordanian universities that offer undergraduate health-related programs (i.e., medicine, dentistry, pharmacy, nursing, or other health-related programs) through official channels and shared with the students via online learning platforms. The survey was open for responses from January 24, 2024, to June 1, 2024. The minimum representative sample size was calculated using the equation below [17]:

 $$\begin{array}{c} \text{Sample size}=\frac{z^{2}\times p\left(1-p\right)/e^{2}}{1+\left(z^{2}\times p\left(1-p\right)/e^{2}N\right)}. \end{array}$$

Given a total population size of 388,678 students enrolled in undergraduate programs, across all disciplines including nonhealthcare programs, among all Jordanian universities (i.e., public and private) [18], with a confidence interval of 95% and a 5% margin of error, the calculated minimum representative sample size was 384, and a total of 465 complete responses were received and included in the study.

The study was reviewed and approved by the institutional review board at the University of Jordan (Approval Number: 158/2024; date: January 23, 2024). The study strictly adhered to the guidelines of the Declaration of Helsinki (1964) on studies involving human subjects with regards to anonymity, voluntary participation, and data protection [19]. In addition, the study subjects did not receive any form of compensation for their participation, and each participant had to submit a consent note prior to revealing the survey questions.

Lastly, the permission to use the Drug Abuse Screening Test (DAST-10) [20] was obtained via email from Prof. Dr. Harvey A. Skinner.

### 2.2. Questionnaire

The questionnaire included four main sections: (1) demographics, (2) attitudes toward substance abuse, (3) the DAST-10, and (4) the prevalence and types of substances of abuse.

The demographics section consisted of 13 questions, including age, gender, marital status, smoking status, work status, living arrangement, type of school attended, type of university attended, current city, program enrolled in, year of study, grade point average (GPA), and if attended a pharmacology course covering drugs affecting the central nervous system (CNS).

The second section involved 12 (yes or no) questions to assess participants' attitudes toward substance abuse, which are as follows: (1) “Do you believe that substance abuse is wrong?”, (2) “Do you believe that substance abusers should be punished?”, (3) “Do you believe that substance abusers need special treatment and professional help?”, (4) “Do you believe that childhood, and how someone was raised contribute to substance abuse?”, (5) “Do you believe that loneliness contributes to substance abuse?”, (6) “Do you believe that friends contribute to substance abuse?”, (7) “Do you believe that individuals with substance abuse issues cannot fully recover?”, (8) “Do you believe that individuals with substance abuse issues do not receive enough support and often neglected by their families, friends or society?”, (9) “As a future healthcare provider, do you believe that you should have a role in preventing substance abuse?”, (10) “Do you believe that healthcare providers are at risk of substance abuse, because they have access to certain medications?”, (11) “Would you still deal with/interact with a healthcare provider with a history of substance abuse?”, and lastly, (12) “Have you ever experienced any suicidal thoughts?”

The third section included the DAST-10 [20], which is copyrighted by Prof. Dr. Harvey A. Skinner. The DAST-10 is a brief self-administered test consisting of 10 (yes or no) questions that provides a quantitative index of the degree of consequences related to drug abuse, and is suitable for adults and older youths. The term “drug use” in DAST-10 refers to “(1) the use of prescribed or over-the-counter drugs in excess of the directions, and (2) any nonmedical use of drugs”. Each yes answer receives 1 point, except for the third question (i.e., no = 1 point), with a possible score range of 0–10. A score of zero means that there is no drug use problem and no action is required, while a score of 9 or 10 suggests a severe degree of problems related to drug abuse and intensive assessment is required. The 10 questions of the DAST-10 tool, which concerns drug use problems within the past 12 months not including alcoholic beverages, were as follows: (1) Have you used drugs other than those required for medical reasons?; (2) Do you use more than one drug at a time?; (3) Are you always able to stop using drugs when you want to? (if never use drugs, answer “yes”); (4) Have you had “blackouts” or “flashbacks” as a result of drug use?; (5) Do you ever feel bad or guilty about your drug use? If never use drugs, choose “no.”; (6) Does your spouse (or parents) ever complain about your involvement with drugs?; (7) Have you neglected your family because of your use of drugs?; (8) Have you engaged in illegal activities in order to obtain drugs?; (9) Have you ever experienced withdrawal symptoms (felt sick) when you stopped taking drugs?; (10) Have you had medical problems as a result of your drug use (e.g., memory loss, hepatitis, convulsions, and bleeding)?

The last part of the questionnaire is aimed at determining the prevalence and types of substances of abuse, with a specific yes or no question “Do you use any illicit drug or substance of abuse?”. Then, the question was followed by a checklist of commonly abused medications and substances to choose from, add other options, or choose nothing for none drug/substance abusers.

### 2.3. Statistical Analysis

Data analysis was conducted using JASP software (Version 0.16.2, www.jasp-stats.org). The results are presented as mean ± standard deviation (SD) or as counts (*n*) and percentages. The dependence across participant demographics, attitudes, and prevalence of substance abuse was assessed using the Chi-square (*χ*^2^) test, except for the continuous variable of age. The normality of distribution of age was tested using the Shapiro–Wilk test, with a significant result (*p* < 0.01) indicating deviation from normal distribution. Accordingly, the participant age ranks were compared using the Mann–Whitney (MW) U test. The effect size for comparisons of means and ranks across dichotomous variables was estimated using Cohen's *d* and rank-biserial correlation (*r*_rb_), respectively. The total DAST-10 score for each participant was calculated using the sum of response scores across all questions, with one question requiring inverse scoring. The DAST-10 score ranks were compared across dichotomous variables using the MW test, while across nominal variables with three or more options through one-way analysis of variance by ranks using the Kruskal–Wallis (KW) test with post hoc Dunn's test using Bonferroni-corrected alpha. The Spearman's rank correlation test was also used to assess the correlations across DAST-10 scores, substance abuse, and demographics, after coding into ordinal variables. Corrections for gender as a confounding variable, using layered classification in Chi-square tests and Spearman's correlation conditioned on gender for DAST-10 scores, were done and are described for corresponding tests in the results section. Lastly, all tests were two-tailed at *α* error of 0.05, and thus, significance was determined at *p* < 0.05.

## 3. Results

### 3.1. Participant Demographics

A total of 465 undergraduate healthcare students, from all 12 governorates of Jordan, participated including males (*n* = 192, 41.3%) and females (*n* = 273, 58.7%). The sample included students of dentistry (31.8%), medicine (26.7%), pharmacy (23.7%), nursing (17%) and a minority of other healthcare programs (0.9%). The sample was collective in terms of year of study, from first- to final-year students, knowledge or lack thereof of the pharmacology of CNS drugs as determined by attendance of its corresponding course, and academic performance, based on GPA rating, from excellent to satisfactory. The mean age of the participants was 21.97 ± 2.1 years, ranging from 18 to 35 years, with higher ranks for males compared to females (*d* = 0.196, *r*_rb_ = 0.118, *p* < 0.05). In terms of demographics, most were single (95.1%), nonsmoking (74.6%), not working (82.2%), living with their families (80%), students of public high schools (52%), and studying at public universities (81.1%). Besides from age, a significant difference based on gender was observed for smoking status, working status, living arrangement, type of university, program and year of study, and GPA ratings (*p* < 0.05). All collected demographics and the impact of gender are summarized in Table 1.

### 3.2. Attitudes Toward Substance Abuse

The next section of the questionnaire covered the attitudes of the participants toward drug/substance abuse and abusers. A total of 11 attitude questions were utilized to investigate their response association with substance abuse among the participants. Most participants believed that substance abuse is wrong (86.2%) and that abusers should be punished (72.3%), need special treatment and professional help (93.6%), and can fully recover (68.8%), but do not receive enough support and are neglected (83.9%). In addition, the majority of the participants agreed that childhood and how a person is raised (90.3%), loneliness (81.1%), and friends (92.7%) are all major contributors to substance abuse. In relation to healthcare, most believe that as future healthcare providers, they should have a role in preventing substance abuse (88.2%), that healthcare providers are at a higher risk of being substance abusers due to access to certain drugs/medications (62.4%) and that they would still interact with a healthcare provider who has a history of substance abuse (58.7%). Following the attitude questions, an additional question was added on whether the participants have previously experienced any suicidal thoughts, with 29.3% answering yes, to assess its potential association with their demographics and drug/substance abuse. Besides age and high school, which had no influence on participant attitudes and experience of suicidal thoughts (*p* > 0.05), all other demographic factors influenced the response distribution of at least one of the questions. The attitude questions and the impact of demographic factors, besides age and high school, are summarized in Table 2.

The nondichotomous demographic factors that influenced the responses were further analyzed to identify the demographic subgroups with higher or lower than expected counts, which were then compared using a chi-square test against all remaining subgroups combined for each factor and question. The results showed the following: (1) higher beliefs that substance abuse is wrong among students who are single (87.3% vs. 65.2%, *p* < 0.01), not working (88% vs. 78.3%, *p* < 0.05), and attending public universities (89.7% vs. 71.6%, *p* < 0.01) as well as those who took a course covering the pharmacology of CNS drugs (89.8% vs. 79.4%, *p* < 0.01), while lower beliefs among pharmacy students (77.3% vs. 89%, *p* < 0.01); (2) the association between the program of study and belief that abusers should be punished was lost with insignificantly higher beliefs among nursing students (79.8% vs. 70.7%, *p* > 0.05); (3) higher beliefs that abusers need special treatment and professional help among students of public universities (95.8% vs. 84.1%, *p* < 0.01) and those who took a course covering the pharmacology of CNS drugs (97.1% vs. 86.9%, *p* < 0.01), while lower beliefs among pharmacy students (86.4% vs. 95.8%, *p* < 0.01) and students living with their friends (80% vs. 94.5%, *p* < 0.01); (4) higher beliefs that childhood and how someone was raised contribute to substance abuse among students who are single (91.4% vs. 69.6%, *p* < 0.01), not working (91.6% vs. 84.3%, *p* < 0.05), and attending public universities (93.6% vs. 76.1%, *p* < 0.01) as well as those who took a course covering the pharmacology of CNS drugs (93.4% vs. 84.4%, *p* < 0.01), while lower beliefs among pharmacy students (79.1% vs. 93.8%, *p* < 0.01); (5) higher beliefs that loneliness contributes to substance abuse among those who live with their families (83.3% vs. 72%, *p* < 0.05), attend public universities (83.3% vs. 71.6%, *p* < 0.05), and took a course covering the pharmacology of CNS drugs (84.6% vs. 74.4%, *p* < 0.01); (6) higher beliefs that friends contribute to substance abuse among students who are not working (94.8% vs. 83.1%, *p* < 0.01), attending public universities (95% vs. 83%, *p* < 0.01), and took a course covering the pharmacology of CNS drugs (95.7% vs. 86.9%, *p* < 0.01), while lower beliefs among pharmacy students (85.5% vs. 94.9%, *p* < 0.01) and those living with their friends (70% vs. 94.3%, *p* < 0.01); (7) higher beliefs that individuals with substance abuse issues cannot fully recover among males (37.5% vs. 26.7%, *p* < 0.05), smokers (41.5% vs. 27.7%, *p* < 0.01), working individuals (43.4% vs. 28.5%, *p* < 0.01), and students of private universities (44.3% vs. 28.1%, *p* < 0.01); however, when corrected for gender, a confounding variable, the influence of smoking and working was lost (*p* > 0.05); (8) higher beliefs that individuals with substance abuse issues do not receive enough support and are often neglected by their families, friends, or society among nonworking (86.4% vs. 72.3%, *p* < 0.01) and public university students (85.9% vs. 75%, *p* < 0.05), while lower beliefs among students living with their friends (60% vs. 85.5%, *p* < 0.01); (9) higher beliefs that, as future healthcare providers, they should have a role in preventing substance abuse among nonworking individuals (90.1% vs. 79.5%, *p* < 0.01), students of public universities (90.7% vs. 77.3%, *p* < 0.01), and those who took a course covering the pharmacology of CNS drugs (93.4% vs. 78.1%, *p* < 0.01), while lower beliefs among pharmacy students (78.2% vs. 91.3%, *p* < 0.01); (10) higher beliefs that healthcare providers are at risk of substance abuse because they have access to certain medications among nursing students (81% vs. 58.6%, *p* < 0.01) and fourth-year students (71.8% vs. 58.2%, *p* < 0.01), while lower among academically poorer-performing students with satisfactory GPA (30% vs. 63.1%, *p* < 0.05 but significance is lost with Yates correction: *p* > 0.05); and (11) no demographic factor had a significant impact on whether the participants would still deal with or interact with a healthcare provider who has a history of substance abuse. In relation to experiencing suicidal thoughts, the response distribution for experiencing them was higher for smoking (37.3% vs. 26.5%, *p* < 0.5), working (38.6% vs. 27.2%, *p* < 0.05), private university (39.8% vs. 26.8%, *p* < 0.05), pharmacy (40.9% vs. 25.6%, *p* < 0.01), and “good” GPA-rating (38.1% vs. 25.7%, *p* < 0.01) students; while lower for students who live with their families (26.6% vs. 39.8%, *p* < 0.05).

### 3.3. Participant DAST-10 Scores

The next section is aimed at screening for and quantifying the degree of drug abuse-related problems among the participants using the DAST-10 questionnaire. The participants scores ranged from 0 to 10, with a mean of 1.6 ± 2.52, and differed by ranks among genders, with males exhibiting higher score ranks (1.92 ± 2.75) than females (1.37 ± 2.33, *p* < 0.05, *d* = 0.219, *r*_rb_ = 0.103). Regarding the degree of problems related to drug abuse, most participants (53.3%) scored 0, indicating no reported problems, followed by a low level (23.4%, score range: 1–2) requiring monitoring and reassessment, a moderate level (11.2%, score range: 3–5) requiring further investigation of the problems, a substantial level (9.5%, score range: 6–8), and a severe level (2.6%, score range: 9–10), with the latter two requiring intensive assessment as the suggested action. Multiple demographic factors influenced the DAST-10 score ranks among the participants, including smoking status, working status, living arrangement, attended university, program of study, year of study, GPA rating, and the attendance of a pharmacology course covering CNS drugs. On the other hand, DAST-10 scores were not correlated with age (*ρ* = 0.06, *p* > 0.05) and were independent of marital status and attended high school (*p* > 0.05). The impact of participant demographics on their DAST-10 scores is summarized in Table 3.

The results showed significantly higher DAST-10 score ranks among students who are smokers (*r*_rb_ = 0.129, *ρ* = 0.106, *p* < 0.05), are working (*r*_rb_ = 0.281, *ρ* = 0.204, *p* < 0.01), attend private universities (*r*_rb_ = 0.475, *ρ* = 0.351, *p* < 0.01), and did not take a course covering the pharmacology of CNS drugs (*r*_rb_ = 0.213, *ρ* = 0.191, *p* < 0.01). The DAST-10 scores were also negatively correlated with GPA (*ρ* = −0.194, *p* < 0.01) and year of study (*ρ* = −0.193, *p* < 0.01), being lower for fifth and sixth year students than third and fourth year students (*p* < 0.01) as revealed by post hoc tests, but with no differences between the first four years of study (*p* > 0.05). In addition, post hoc tests revealed higher DAST-10 score ranks for students living with their friends rather than alone or with their families (*p* < 0.01) and pharmacy students compared to medicine, nursing, and dentistry students (*p* < 0.01), with the latter, dentistry students, having lower scores than medicine and nursing students as well (*p* < 0.05). Upon correction for gender, the relationship between DAST-10 scores and smoking was lost (*ρ* = 0.08, *p* > 0.05), but remained significant with working status (*ρ* = 0.189, *p* < 0.01), attended university (*ρ* = 0.345, *p* < 0.01), GPA (*ρ* = −0.188, *p* < 0.01), and year of study (*ρ* = −0.198, *p* < 0.01). In relation to participant attitudes, the DAST-10 scores were significantly lower for students who believe that substance abuse is wrong (*d* = 0.762, *r*_rb_ = 0.391, *p* < 0.01), abusers need special treatment (*d* = 0.943, *r*_rb_ = 0.543, *p* < 0.01), childhood contributes to substance abuse (*d* = 0.77, *r*_rb_ = 0.432, *p* < 0.01), loneliness contributes to substance abuse (*d* = 0.14, *r*_rb_ = 0.128, *p* < 0.05), friends contribute to substance abuse (*d* = 0.859, *r*_rb_ = 0.482, *p* < 0.01), substance abusers can fully recover (*d* = 0.536, *r*_rb_ = 0.312, *p* < 0.01), substance abusers do not receive enough support (*d* = 0.33, *r*_rb_ = 0.209, *p* < 0.01), and they should have a role in preventing substance abuse as future healthcare providers (*d* = 0.66, *r*_rb_ = 0.403, *p* < 0.01). Regarding suicidal thoughts, the students who reported previously experiencing them had significantly higher DAST-10 score ranks (2.88 ± 3.1) than the students who did not experience them (1.07 ± 2.02, *d* = 0.756, *d* = 0.756, *r*_rb_ = 0.345, *p* < 0.01).

### 3.4. Prevalence of Substance Abuse

The last section of the questionnaire is aimed at assessing the prevalence of substance abuse among the participants, evaluating its association with demographics, attitudes, and DAST-10 scores, and identifying the classes of drugs/substances of abuse among the participants. A total of 64 students (13.76%) reported the use of at least one substance of abuse. In terms of demographics, five factors were associated with substance abuse: being a smoker (19.5% vs. 11.82%, OR: 1.81, 95% CI: 1.03–3.16, *p* < 0.05), working in addition to studying (21.7% vs. 12%, OR: 2.02, 95% CI: 1.1–3.71, *p* < 0.05), attending private universities (21.6% vs. 11.9%, OR: 2.03, 95% CI: 1.12–3.69, *p* < 0.05), being a pharmacy student (30.9% vs. 8.5%, OR: 4.85, 95% CI: 2.79–8.41, *p* < 0.01), and being a second year student (30.4% vs. 11.9%, OR: 3.23, 95% CI: 1.61–6.46, *p* < 0.01). On the other hand, no association was observed (*p* > 0.05) between substance abuse and gender, age, marital status, living arrangement, high school, GPA, and attending a course covering the pharmacology of CNS drugs. Regarding participant attitudes, five factors were also associated with substance abuse: not believing that substance abusers should be punished (19.4% vs. 11.6%, OR: 1.83, 95% CI: 1.06–3.17, *p* < 0.05), not believing that childhood and how someone was raised contribute to substance abuse (24.4% vs. 12.6%, OR: 2.24, 95% CI: 1.07–4.69, *p* < 0.05), not believing that friends contribute to substance abuse (26.5% vs. 12.8%, OR: 2.46, 95% CI: 1.09–5.55, *p* < 0.05), believing that substance abusers cannot fully recover (22.1% vs. 10%, OR: 2.55, 95% CI: 1.49–4.36, *p* < 0.01), and still wanting to deal or interact with a healthcare provider who has a history of substance abuse (16.5% vs. 9.9%, *p* < 0.05) but with insignificant odds (OR: 1.8, 95% CI: 1.02–3.18, *p* > 0.05). The experience of suicidal thoughts was also found associated with substance abuse (25% vs. 9.1%, OR: 3.32, 95% CI: 1.94–5.7, *p* < 0.01). Next, the correlation between DAST-10 scores and substance abuse was assessed; indeed, a moderate and significant positive correlation was observed (*ρ* = 0.442, *p* < 0.01), and a very significant difference in DAST-10 scores was revealed between individuals who use substances of abuse (4.52 ± 3.07) and those who do not (1.14 ± 2.07, *d* = 1.51, *r*_rb_ = 0.679, *p* < 0.01). Lastly, the substances of abuse reported by the participants were classified into seven major classes to assess their rates of usage. The main reported class was benzodiazepines (22.6%), followed by antidepressants (19.8%), CNS stimulants (18.7%), cannabis products and preparations (11.1%), antiepileptic drugs (10.7%), opioids and derivatives (9.5%), and lastly hallucinogens other than cannabis (7.5%).

## 4. Discussion

This study offers a comprehensive analysis of substance abuse among undergraduate healthcare students in Jordan. The results revealed a relatively high prevalence of substance abuse among the students, which aligns with recent findings on university students in Jordan [21], and identified key demographics, attitudinal factors, and beliefs associated with this behavior. The findings could aid future efforts in developing and implementing interventional strategies to mitigate the issue of substance abuse among healthcare students.

In terms of demographics, several factors were found to significantly associate with substance abuse: being a smoker, working while studying, attending private universities, being a pharmacy student, and being in the second year of study. These correlations mirror findings from various studies. For example, a study in the United States indicated that individuals who smoked cigarettes were more likely to engage in substance abuse, suggesting that cigarette smoking may act as a gateway to illegal drug use [22]. This is also supported by the comorbidity between tobacco smoking and substance abuse and relapse [23, 24]. Additionally, substance use was significantly associated with students working while studying, as observed in a study from Egypt [3]. This association was observed even for school students decades ago [25] with many potential explanations and contributing factors including extra money availability and coexistence of mental health problems. The higher prevalence of substance abuse among private university students is also consistent with recent findings in Jordan, showing that studying in public universities acts as a protective factor against substance abuse [26]. Similarly, the higher prevalence of substance abuse among pharmacy students is supported by research showing elevated rates of illegal drug use among students in pharmacy compared to nursing programs in Nepal [2]. Being in the second year of study aligns more with findings from Jordan indicating that most students who use drugs of abuse start young, at around 18 years of age [26]; however, it is contrary to other findings from Egypt showing that substance abuse was more common among final-year students [3].

Regarding participant attitudes, five factors were significantly associated with higher rates of abuse: not believing that substance abusers should be punished, not believing that childhood and how someone was raised contribute to substance abuse, not believing that friends contribute to substance abuse, believing that substance abusers cannot fully recover, and still wanting to deal or interact with a healthcare provider who has a history of substance abuse. These attitudes of participants who use substances of abuse, except the latter, were contrary to the beliefs of most participants. Indeed, most participants believed that abusers should be punished, despite that treatment should be emphasized over punishment [27], that friends and childhood contribute to abuse, which are evident and well-established associations [28, 29], and that substance abusers can fully recover. Although most participants would still interact with a healthcare provider who has a history of substance abuse, the ratio among substance abusers was significantly higher than nonabusers, which reflects how stigma toward substance abuse can affect trust in healthcare providers, which is a crucial factor in patient interactions. These findings further signify the deviation of attitudes among substance abusers, especially regarding the lack of awareness of influencing factors such as friends and their negative perceptions of being unable to recover, which is linked to anxiety and depression which can lead to relapse [30], consistent with the observed association between substance abuse and experiencing suicidal thoughts, which also is evident in the literature [31]. Accordingly, the findings support that cognitive therapy and changing perceptions could play pivotal roles in managing substance abuse [30]. This in addition to the important need for increased education and awareness [32].

The DAST-10 scale results were found to correlate with actual abuse among the participants and many of its associated factors, such as smoking, working, attending private universities, studying pharmacy, and experiencing suicidal thoughts. Accordingly, the findings support the usefulness of this scale as a screening tool for substance abuse problems among healthcare students in Jordan. However, DAST-10 scores are associated with additional demographic factors and attitudes compared to actual abuse, which may provide further insights into additional risk determinants. For instance, higher DAST-10 scores were observed for males, living with friends, and lower academic performance in terms of GPA. Additional noteworthy observations are the lower DAST-10 scores associated with the belief that substance abuse is wrong, which is reported as a major determinant for preventing substance abuse and maintaining abstinence among adolescent students [33], and with attending a pharmacology course covering the pharmacology of CNS drugs, supported by reports on how the lack of education of medical students on substance abuse may lead to informal and potentially dangerous learning experiences [34], especially considering that the most reported class of drugs of abuse was benzodiazepines.

Lastly, it should be noted that one limitation of the study is the potential for self-selection bias. Although the questionnaire was distributed to all Jordanian universities offering undergraduate health-related programs, participation was voluntary and based on self-selection. Accordingly, this may limit the generalizability of the current findings.

## 5. Conclusions

In conclusion, results of the current work shed light on the relatively high prevalence of substance abuse and its associated factors among healthcare students in Jordan. This, in turn, necessitates the implementation of interventional strategies by relevant parties and policy makers to mitigate substance abuse and increase awareness in this critical group to prevent later consequences given their future role as public health providers.

## Figures and Tables

**Table 1:** Participant demographics.

**Variable** ^**a**^	**Total**	**Males**	**Females**	**p** ** value** ^b^
Age	21.97 (2.1)	22.21 (2.15)	21.8 (2.04)	0.028⁣^∗^
*Marital status*				0.648
Single	442 (95.1)	183 (95.3)	259 (94.9)	
Engaged	8 (1.7)	3 (1.6)	5 (1.8)	
Married	14 (3)	5 (2.6)	9 (3.3)	
Other	1 (0.2)	1 (0.5)	0 (0)	
*Smoking status*				< 0.001⁣^∗^
Nonsmoking	347 (74.6)	107 (55.7)	240 (87.9)	
Smoking	118 (25.4)	85 (44.3)	33 (12.1)	
*Working status*				< 0.001⁣^∗^
Studying only	382 (82.2)	140 (72.9)	242 (88.6)	
Working and studying	83 (17.8)	52 (27.1)	31 (11.4)	
*Living arrangement*				< 0.001⁣^∗^
Alone	63 (13.6)	31 (16.2)	32 (11.7)	
With family	372 (80)	140 (72.9)	232 (85)	
With friends	30 (6.4)	21 (10.9)	9 (3.3)	
*High school*				0.51
Military	3 (0.7)	1 (0.5)	2 (0.7)	
Private	220 (47.3)	85 (44.3)	135 (49.5)	
Public	242 (52)	106 (55.2)	136 (49.8)	
*University*				0.037⁣^∗^
Private	88 (18.9)	45 (23.4)	43 (15.8)	
Public	377 (81.1)	147 (76.6)	230 (84.2)	
*Study program*				0.02⁣^∗^
Dentistry	148 (31.8)	51 (26.6)	97 (35.5)	
Medicine	124 (26.7)	62 (32.3)	62 (22.7)	
Nursing	79 (17)	26 (13.5)	53 (19.4)	
Pharmacy	110 (23.6)	50 (26)	60 (22)	
Other	4 (0.9)	3 (1.6)	1 (0.4)	
*Year of study*				0.026⁣^∗^
1^st^	15 (3.2)	6 (3.1)	9 (3.3)	
2^nd^	46 (9.9)	13 (6.8)	33 (12.1)	
3^rd^	88 (18.9)	37 (19.3)	51 (18.7)	
4^th^	142 (30.5)	67 (34.9)	75 (27.5)	
5^th^	143 (30.8)	50 (26)	93 (34)	
6^th^	31 (6.7)	19 (9.9)	12 (4.4)	
*GPA rating*				0.027⁣^∗^
Excellent	123 (26.5)	51 (26.6)	72 (26.4)	
Very good	198 (42.6)	68 (35.4)	130 (47.6)	
Good	134 (28.8)	68 (35.4)	66 (24.2)	
Satisfactory	10 (2.1)	5 (2.6)	5 (1.8)	
*Attended pharmacology course covering CNS drugs*				0.543
No	160 (34.4)	63 (32.8)	97 (35.5)	
Yes	305 (65.6)	129 (67.2)	176 (64.5)	

^a^Data is presented as *n* (%), except for age as mean (SD).

^b^Chi-square test except age (MW test).

⁣^∗^Significant (*p* < 0.05).

**Table 2:** The association between participant demographics and their attitudes toward substance abuse and experience of suicidal thoughts.

**Impact of participant demographics** ^**a**^, **p values**^**b**^									
**Gender**	**Marital status**	**Smoking status**	**Working status**	**Living arrangement**	**University**	**Program**	**Year**	**GPA**	**Attended course** ^**c**^
Q1. Do you believe that substance abuse is wrong?									
0.226	0.01⁣^∗^	0.393	0.021⁣^∗^	0.074	< 0.001⁣^∗^	0.034⁣^∗^	0.339	0.087	0.002⁣^∗^
Q2. Do you believe that substance abusers should be punished?									
0.104	0.45	0.136	0.593	0.58	0.709	0.009⁣^∗^	0.9	0.733	0.894
Q3. Do you believe that substance abusers need special treatment and professional help?									
0.814	0.644	0.484	0.072	0.007⁣^∗^	< 0.001⁣^∗^	0.005⁣^∗^	0.77	0.205	< 0.001⁣^∗^
Q4. Do you believe that childhood, and how someone was raised contribute to substance abuse?									
0.853	0.003⁣^∗^	0.609	0.042⁣^∗^	0.35	< 0.001⁣^∗^	< 0.001⁣^∗^	0.501	0.111	0.002⁣^∗^
Q5. Do you believe that loneliness contributes to substance abuse?									
0.689	0.88	0.928	0.102	0.015⁣^∗^	0.012⁣^∗^	0.115	0.419	0.256	0.008⁣^∗^
Q6. Do you believe that friends contribute to substance abuse?									
0.284	0.867	0.332	< 0.001⁣^∗^	< 0.001⁣^∗^	< 0.001⁣^∗^	0.004⁣^∗^	0.148	0.16	< 0.001⁣^∗^
Q7. Do you believe that individuals with substance abuse issues cannot fully recover?									
0.014⁣^∗^	0.737	0.005⁣^∗^	0.008⁣^∗^	0.069	0.003⁣^∗^	0.181	0.21	0.116	0.134
Q8. Do you believe that individuals with substance abuse issues do not receive enough support and often neglected by their families, friends or society?									
0.792	0.343	0.15	0.002⁣^∗^	0.001⁣^∗^	0.012⁣^∗^	0.283	0.62	0.603	0.1
Q9. As a future healthcare provider, do you believe that you should have a role in preventing substance abuse?									
0.096	0.141	0.329	0.007⁣^∗^	0.121	< 0.001⁣^∗^	0.004⁣^∗^	0.421	0.183	< 0.001⁣^∗^
Q10. Do you believe that healthcare providers are at risk of substance abuse, because they have access to certain medications?									
0.526	0.437	0.159	0.576	0.355	0.446	0.001⁣^∗^	0.008⁣^∗^	0.028⁣^∗^	0.244
Q11. Would you still deal with/interact with a healthcare provider with a history of substance abuse?									
0.164	0.51	0.215	0.671	0.596	0.748	0.238	0.094	0.054	0.435
Q12. Have you ever experienced any suicidal thoughts?									
0.811	0.214	0.026⁣^∗^	0.04⁣^∗^	0.044⁣^∗^	0.016⁣^∗^	0.019⁣^∗^	0.578	0.018⁣^∗^	0.965

^a^Age and high school were omitted as they had no impact on any response (*p* > 0.05).

^b^Chi-square test.

^c^Pharmacology course covering CNS drugs.

⁣^∗^Significant (*p* < 0.05).

**Table 3:** Impact of participant demographic factors on DAST-10 score ranks.

**Variable** ^**a**^	**DAST-10 score** **Mean (SD)**	**p** ** value** ^**b**^
*Gender*		0.039⁣^∗^
Male	1.92 (2.75)	
Female	1.37 (2.33)	
*Working status*		< 0.001⁣^∗^
Studying only	1.41 (2.44)	
Working and studying	2.47 (2.72)	
*Marital status*		0.435
Single	1.58 (2.48)	
Engaged	1.38 (3.11)	
Married	2.57 (3.28)	
Other	0 (−)^c^	
*Living arrangement*		0.005⁣^∗^
Alone	1.79 (2.79)	
With family	1.43 (2.37)	
With friends	3.30 (3.08)	
*Study program*		< 0.001⁣^∗^
Dentistry	0.63 (1.45)	
Medicine	1.63 (2.68)	
Nursing	1.61 (2.73)	
Pharmacy	2.84 (2.76)	
Other	2.5 (3.11)	
*Attended pharmacology course covering CNS drugs*		< 0.001⁣^∗^
No	2.19 (2.8)	
Yes	1.29 (2.31)	
*Smoking status*		0.023⁣^∗^
Nonsmoking	1.45 (2.36)	
Smoking	2.05 (2.89)	
*University*		< 0.001⁣^∗^
Private	3.15 (2.88)	
Public	1.24 (2.29)	
*GPA rating*		< 0.001⁣^∗^
Excellent	0.95 (2.0)	
Very good	1.51 (2.3)	
Good	2.36 (3.05)	
Satisfactory	1.3 (2.26)	
*High school*		0.051
Military	2.33 (3.22)	
Private	1.34 (2.36)	
Public	1.83 (2.64)	
*Year of study*		< 0.001⁣^∗^
1^st^	1.87 (2.7)	
2^nd^	1.61 (2.3)	
3^rd^	1.91 (2.54)	
4^th^	2.16 (2.97)	
5^th^	1.03 (2.0)	
6^th^	0.65 (1.8)	

^a^Age was omitted (Spearman's correlation: *p* = 0.204).

^b^MW test for dichotomous and KW test for nondichotomous variables.

^c^ *n* = 1.

⁣^∗^Significant (*p* < 0.05).

## Data Availability

The data that support the findings of this study are available from the corresponding author upon reasonable request.
